# Effects of Exposure to the Sound from Seismic Airguns on Pallid Sturgeon and Paddlefish

**DOI:** 10.1371/journal.pone.0159486

**Published:** 2016-08-09

**Authors:** Arthur N. Popper, Jackson A. Gross, Thomas J. Carlson, John Skalski, John V. Young, Anthony D. Hawkins, David Zeddies

**Affiliations:** 1 Department of Biology, University of Maryland, College Park, Maryland, United States of America; 2 Smith – Root Inc., Vancouver, Washington, United States of America; 3 ProBioSound, LLC, Holmes Beach, Florida, United States of America; 4 University of Washington, School of Aquatic & Fishery Sciences, Seattle, Washington, United States of America; 5 CSA Ocean Sciences Inc., Stuart, Florida, United States of America; 6 Loughine Ltd, Kincraig, Blairs, Aberdeen, United Kingdom; 7 JASCO Applied Sciences Ltd., Washington, D.C., United States of America; Pacific Northwest National Laboratory, UNITED STATES

## Abstract

This study examined the effects of exposure to a single acoustic pulse from a seismic airgun array on caged endangered pallid sturgeon (*Scaphirhynchus albus*) and on paddlefish (*Polyodon spathula*) in Lake Sakakawea (North Dakota, USA). The experiment was designed to detect the onset of physiological responses including minor to mortal injuries. Experimental fish were held in cages as close as 1 to 3 m from the guns where peak negative sound pressure levels (Peak- SPL) reached 231 dB re 1 μPa (205 dB re 1 μPa^2^·s sound exposure level [SEL]). Additional cages were placed at greater distances in an attempt to develop a dose-response relationship. Treatment and control fish were then monitored for seven days, euthanized, and necropsied to determine injuries. Necropsy results indicated that the probability of delayed mortality associated with pulse pressure following the seven day monitoring period was the same for exposed and control fish of both species. Exposure to a single pulse from a small air gun array (10,160 cm^3^) was not lethal for pallid sturgeon and paddlefish. However, the risks from exposure to multiple sounds and to sound exposure levels that exceed those reported here remain to be examined.

## Introduction

While there is growing interest in the potential impact of man-made (anthropogenic) sounds on aquatic organisms, very little is known about the effects of high-intensity sound exposure on fishes (e.g., [1–4], including those from seismic airguns used in exploration for gas and oil. While the few studies that have been performed have shown no mortal injuries to fishes as a result of airgun exposure, the paucity of data and the variation in experimental approaches, as well as the wide diversity in physiology, behavior, and anatomy of different species, suggests that further work is needed to understand fully the effects of seismic airguns on fishes [1, 3].

A number of behavioral studies have examined the effects of seismic airgun sounds on fish behavior. Various studies have shown that some species will avoid seismic sounds [5–8], while other studies with other species have shown no response [9–11]. The variability in the response data could be related to numerous factors including, but not limited to: species studied, time of year, intensity of the airgun source, and even motivation of the fish at the time of exposure in terms of how they respond to potentially noxious stimuli [11]. Indeed, a recent study used a simulated pile driving sound as the source (a source that is relatively similar to that of an airgun) and showed that although two schooling species (sprat *Sprattus sprattus* and mackerel *Scomber scombrus*) responded during the day to moderate sound pressure levels (163.2 and 163.3 dB re 1 μPa peak-to-peak received levels, respectively), neither species responded even to the highest sound levels presented at night when the schools had dispersed [12].

A small number of studies have examined physiological effects of exposure to other impulsive sound sources on fishes including simulated pile driving sounds [13–18] and seismic water guns [19]. The highly quantified pile driving studies by Halvorsen et al. [17, 18] and Casper et al. [13, 15, 16] examined physiological effects on several different species and demonstrated a general dose-response effect as cumulative sound exposure level (SELcum—defined as the accumulated sound exposure of the combined pulses to which the fish were exposed [20, 21]) increased. Onset of physiological effects started with exposure to an SEL_cum_ of around 207 dB re 1 μPa^2^ s (depending on the species) with small hematomas in the skin and reached maximum effects, with likely mortality, at an SEL_cum_ of approximately 210–216 dB re 1 μPa2 s. Swim bladder rupture, when encountered, was not seen until the SEL_cum_ was 216 dB re 1 μPa^2^·s or greater.

Gross et al. [19] exposed fish to two acoustic pulses from a 5,621 cm^3^ seismic watergun. In this study, 87% of the northern pike (*Esox lucius*) (50.9 ± 12.6 cm mean total length ± sd) showed swim bladder ruptures when fish were 9 m from the source gun with a received single strike sound exposure level (SEL_ss_) of 199.5 dB re 1 μPa^2^·s. The differences in onset of swim bladder rupture between the pile driving and water gun studies are not clear at this point, but could be related to species, fish size, and the differences in onset or duration between pile driving and seismic sounds. Indeed, water-gun studies may not be directly applicable to results from airguns due to differences in the acoustic signatures and signal durations of the two devices [22].

The purpose of the present study was to examine the effects of exposure to seismic air guns on pallid sturgeon (*Scaphirhynchus albus*) and paddlefish (*Polyodon spathula*). In addition, a goal of the study was to enable development of a dose-response function whereby the levels of sound energy received by fishes at different distances from the source could be quantitatively related to the response of the fishes to the sound exposure. The sound stimulus was from a seismic airgun array such as those being used in oil and gas exploration throughout the upper Missouri River basin (USA). The general paradigm in such studies is to move an airgun array along a grid, stopping at set distances (often about 100 m), triggering the airguns, and then moving to the next point on the grid.

## Methods

### Study Site

**Fig 1:**
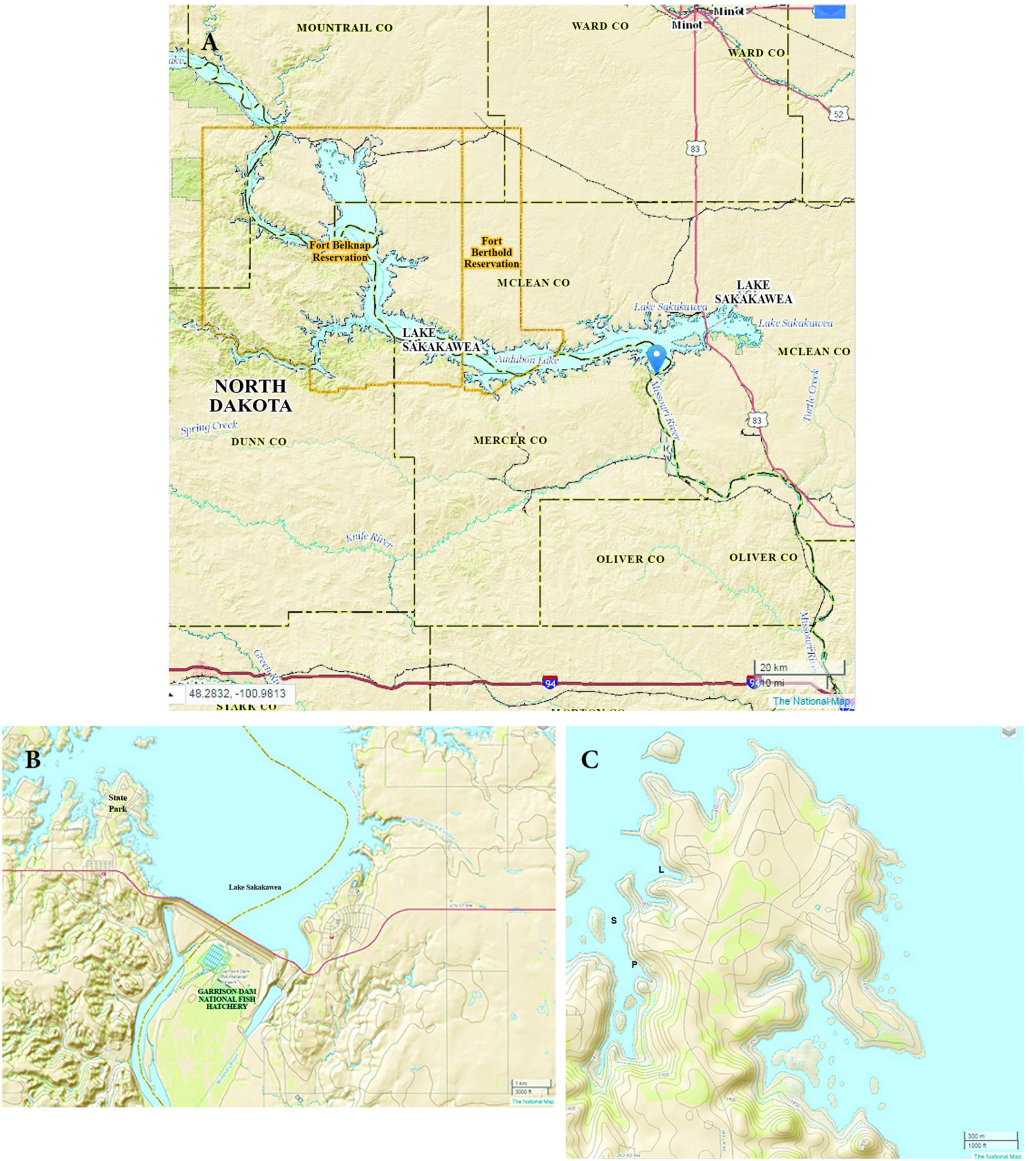
Study Sites. A: Lake Sakakawea with blue dot showing site of study. B. The study site from (A) is shown enlarged, including the Garrison Dam and the fish hatchery at which the fish were raised and held. C. Lake Sakakawea State park showing the study site (S) and boat anchorages (L—boat launch site; P—Pontoon boat launch site). Images from NationalMap.Gov of the U.S. Department of Geological Survey.

The study site was on the west side of Lake Sakakawea State Park (Fig 1), which is located on the south side of the eastern end of Lake Sakakawea near Park City, North Dakota (USA). The fish were subsequently held, and necropsy performed, at the Garrison Dam National Fish Hatchery (GDNFH) which is located approximately 1.6 km east-southeast of the park.

The site for the fish exposure study was chosen for its relative proximity to the GDNFH and thus ease of moving animals to the lake, the availability of a staging area for the study (including a boat ramp), and because it was an area with low likelihood of occurrence of wild pallid sturgeon and paddlefish so as not to potentially impact non-experimental animals.

### Study Species

Three-year-old pallid sturgeon and two-year-old paddlefish had been hatched and reared at GDNFH for this study. Only fish that were within one standard deviation from the mean length of each test population were used (Table 1). Pallid sturgeon and paddlefish were held together at the hatchery in 1.8 m diameter circular, black fiberglass tanks. Water for the hatchery was provided by ambient lake water with a temperature of 14°C during the experimental weeks from September 6 to 23, 2012. All aspects of the animal use on this project including maintenance, euthanasia, necropsy, and actual experiments were done following the Guidelines the Use of Fishes in Research of the American Fisheries Society (http://fisheries.org/docs/wp/Guidelines-for-Use-of-Fishes.pdf) and under appropriate permits from the United States Fish and Wildlife Service.

**Table 1:** Number of Fish Exposed or Used as Controls.

Species	Number of Fish Used (exposed and controls)	Mean Fish Length (mm ± SD)	Mean Fish Weight (g ± SD)
Pallid Sturgeon	90	414 ± 25	224 ± 63
Paddlefish	71	468 ± 17	352 ± 44

SD = standard deviation.

### Identification of Individual Fish

Fish were individually marked 6 to 8 days before sound exposure. All fish were handled the same way and without sedation. Tagging involved fish being taken individually from holding tanks using a dip net, measured (fork length), tagged, and then being placed in a separate tank that held only tagged fish. Tag numbers were recorded along with fish length. Pallid sturgeon were implanted with a 12 mm passive integrated transponder (PIT) tag (Biomark, Boise ID USA), while paddle fish received Floy T-bar anchor tags (Floy Tag, Seattle, Washington USA). Each individually numbered tag was placed in the dorsal musculature posterior and lateral to the dorsal fin.

### Fish Cages and Transportation

**Fig 2:**
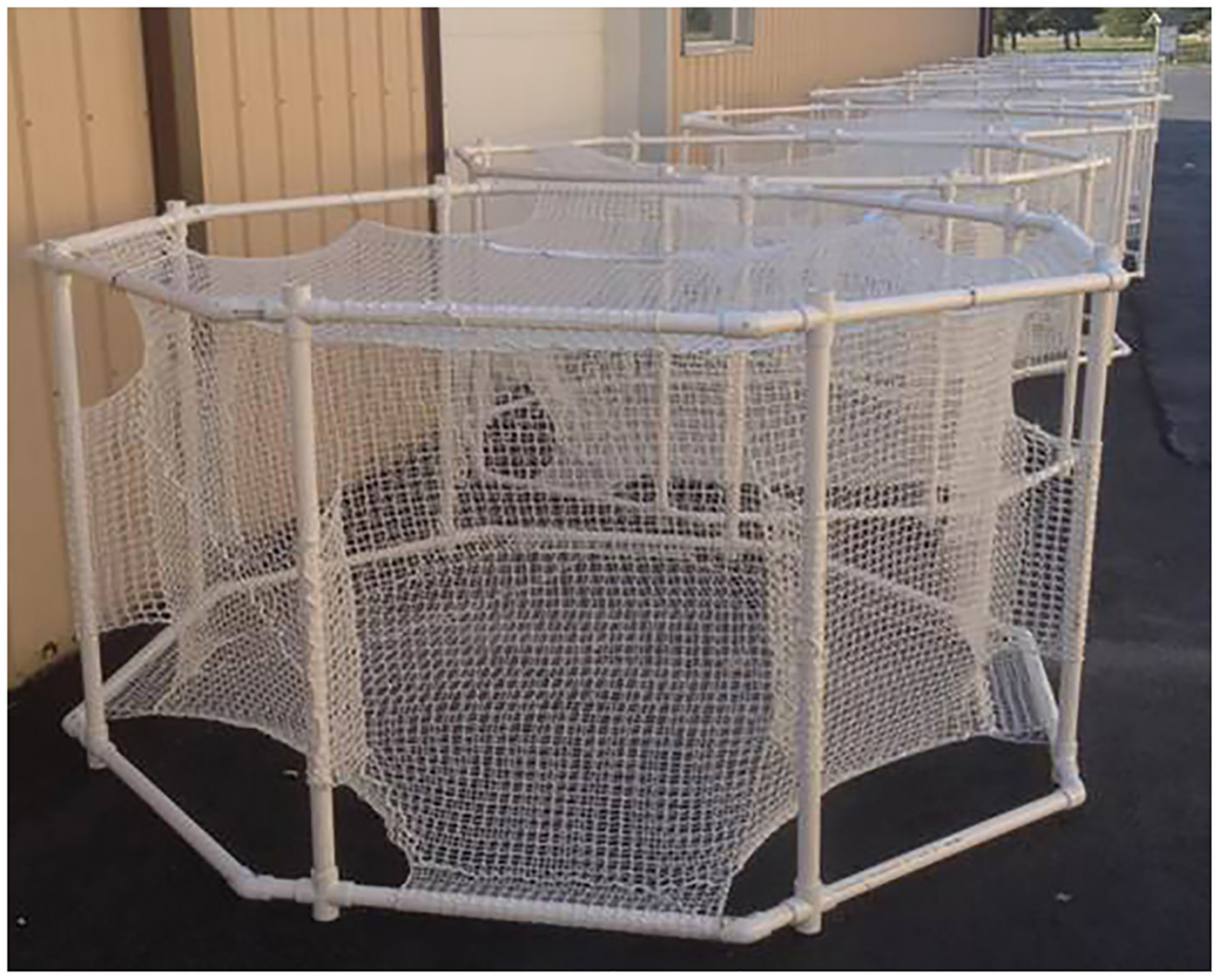
Fish exposure cages.

Fish exposure cages (Fig 2) were constructed of 2.54-cm square braided knotless mesh mounted in a frame constructed of 2.54-cm polyvinyl chloride (PVC) pipe. The mesh cages (Miller Net Company, Memphis TN USA) were 1 m high x 1.5 m wide and were designed to: 1) keep all fish as close as possible to the center of the cage so that all were exposed to the same signal level; 2) provide ample swimming space for up to five fish per cage (though fewer were always used); 3) reduce the risk of entanglement or injury to fish from the mesh or hard frame; and 4) allow for continuous swimming with an octagonal-shaped cage (no right angles) because paddlefish and sturgeon have rather inflexible bodies and cannot easily turn. In static water both species must be able to swim continuously so as to provide movement of water across gill membranes for respiration.

Once the transport truck arrived at the marina boat ramp the fish were transferred by dip net into one of three rectangular aluminum cattle troughs on the pontoon barge for transport to the test site (Fig 1c). Water temperature and dissolved oxygen levels were monitored during the temperature acclimation process and recorded approximately every 30 min.

All fish were held on the transport barge for the duration of the experiment except when they were moved to the test cages for exposure to sound. At the completion of all sound exposures on a given day the fish were returned to the boat ramp using the pontoon barge. The fish were then transferred to the empty tank on the haul trailer and immediately returned to the hatchery.

Once back at the hatchery, fish of each species were transferred to separate1.83 m x 2.4 m oval black fiberglass tanks (11.5 m^3^). Fish tanks were monitored every 12 h for dissolved oxygen and fish mortality. Feeding of the test fish was stopped the day before tagging and was not resumed for the duration of the study.

### Controls

Controls were treated precisely as the experimental animals, including being submerged in cages for the same time period. The only difference was that these fishes received no sound exposure.

### Experimental Procedure

**Fig 3:**
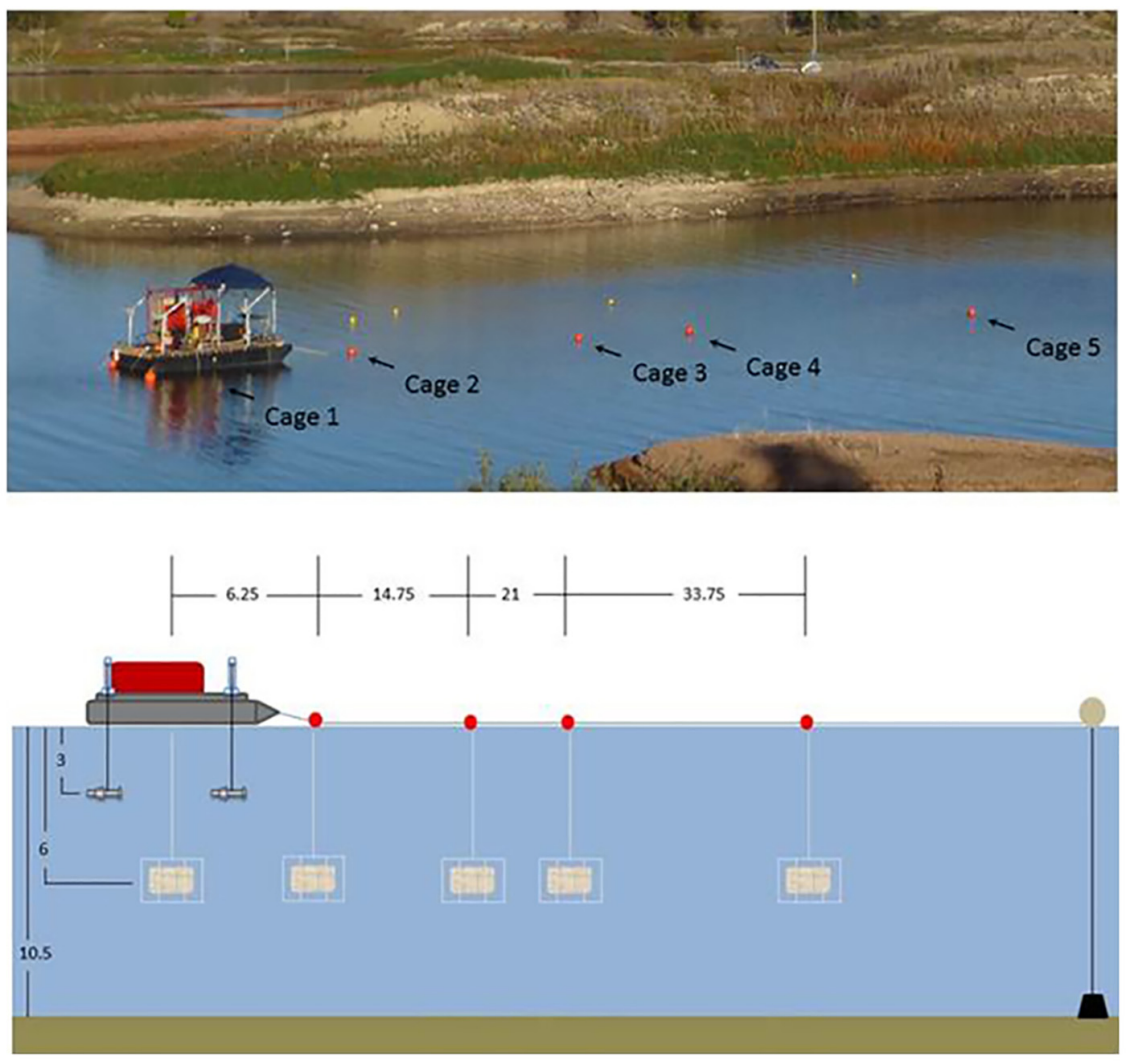
Airgun barge and fish exposure cage locations in Lake Sakakawea. **Top**: Photograph of the experimental setup. The seismic barge is to the left. Red floats indicate cage and autonomous multichannel acoustic recorder (AMAR) locations. Yellow floats are surface floats used for AMAR retrieval (they do not indicate the location of the AMARs). Airguns were hung from davits near the corners of the barge. The control cage is not shown in this figure, but it would be to the left (south) of the airgun barge. **Bottom**: Schematic of the locations of the five exposure cages relative to the airgun barge (upper left) and the airgun array (just below the barge). Distances in meters. Figure shows exposure cages at a depth of 6 m for pallid sturgeon. For paddlefish, the exposure cages were at a depth of 2 m (Table 2).

The experiment design was randomized block to control for temporal effects over the course of the exposure trials. A block, defined as a single replicate at each of the six exposure locations, consisted of exposing a set of fish in cages numbered 1 to 5 plus a control cage in a random sequence. The 5 sound exposure cages were located at varying distance measured from the center of the seismic airgun array (Fig 3). A sixth (control) cage was placed about 150 m south of the seismic array. The order of exposure location (cage) within each block of exposures was determined prior to testing using a random number generator. Once a block was finished, another block was run using a different random sequence. The number of replicates (blocks) and fish exposed in each cage is indicated in Table 2.

**Table 2:** Fish Exposure Information.

Species	Number of Replicates	Number of Fish Exposed Per Cage	Cage Depth*	Time from Placement on Pontoon Boat to Return to Haul Trailer (average)
Pallid sturgeon	5	3	6 m	4.33 h
Paddlefish	3	4	2 m	4.25 h

* Cage depth is the water depth of the cage measured from the vertical center of the cage.

In each experimental replicate (block) fish were placed in each of five sound exposure cages and the control cage. Fish placed in the control cage were treated identically to those in the sound exposure cages, except that the airgun array was not discharged when control fish were in the water. The cages were at 6 m depth for pallid sturgeon and at 2 m for paddlefish since they tend to live closer to the water surface than pallid sturgeon. As they were placed at different depths, the two species were tested on different days.

The experimental paradigm involved exposing each animal to sound only once. Groups of fish of one or the other species were placed in cages at various distances from the source, resulting in exposure to different sound levels.

The specific procedure started with fish being randomly selected from the transport barge, placed on an aluminum boat in a 189 L tub with fresh aerated lake water, transported to the exposure area and dip netted into the exposure cage. Once loaded with three or four fish, the cage was lowered to the depth designated for the particular species (Table 2), the compressor was activated to charge the airguns to their operating pressure. Sixty seconds after the operating air pressure was reached, the airgun array was triggered to expose the fish to a single seismic pulse. The boat then returned to the cage location, the cage was raised to the surface, and the fish were removed using a dip net and then put into a tub, taken to the pontoon transport barge, and then placed into a receiving trough. The average time from the time the fish was removed from the trough to its return after an exposure was about 10 min.

In each experimental replicate (one study day), fish were placed in each of five cages located at varying distance measured from the center of the seismic airgun array (Fig 3). In addition, a sixth control cage was placed about 150 m south of the array. Fish placed in the control cage were treated identically to those in the sound exposure cages, except that the airgun array was not discharged when control fish were in the water. The cages were at 6 m depth for pallid sturgeon and at 2 m for paddlefish since they tend to live closer to the water surface than pallid sturgeon. As they were placed at different depths, the two species were tested on different days.

By exposing only one cage at a time it was possible to ensure that all fish were treated consistently and that all spent the same amount of time at depth before being exposed to airgun sounds. It should be noted that the physiological condition of fish at the time of exposure, including whether the swim bladder was fully inflated at depth, was unknown, other than that the fish were active and appeared healthy before being lowered to depth and they were active and appeared healthy when returned to the surface and placed in the holding tank on the transport boar.

### Airgun Barge

The airgun barge (Fig 3A) was outfitted with four Bolt Technologies Incorporated (Norwalk, Connecticut, USA) Long Life airguns that were lowered to a depth of 3m. Three airguns had a volume of 2,294 cm^3^ while one was 3,277 cm^3^, totaling 10,160 cm^3^. Air pressure for each airgun, 13,789.5 kPa, was achieved using a high pressure compressor rated to 100 cfm @ 34,473 kPa (Stark Industries, Houston, Texas, USA). Davits were mounted in a rectangular configuration, 2.75 m wide by 3.7 m long and the four airguns were raised and lowered to a depth of 3 m. All four airguns were triggered simultaneously.

### Acoustic Methodology

Sound exposure data were obtained using a combination of real-time and autonomous recording systems to measure received levels at the cages before and during the study and at a remote control location. These received levels were used to correlate the effects on the fish (e.g., immediate or delayed mortality) with the dose (sound) received by the fish.

**Fig 4:**
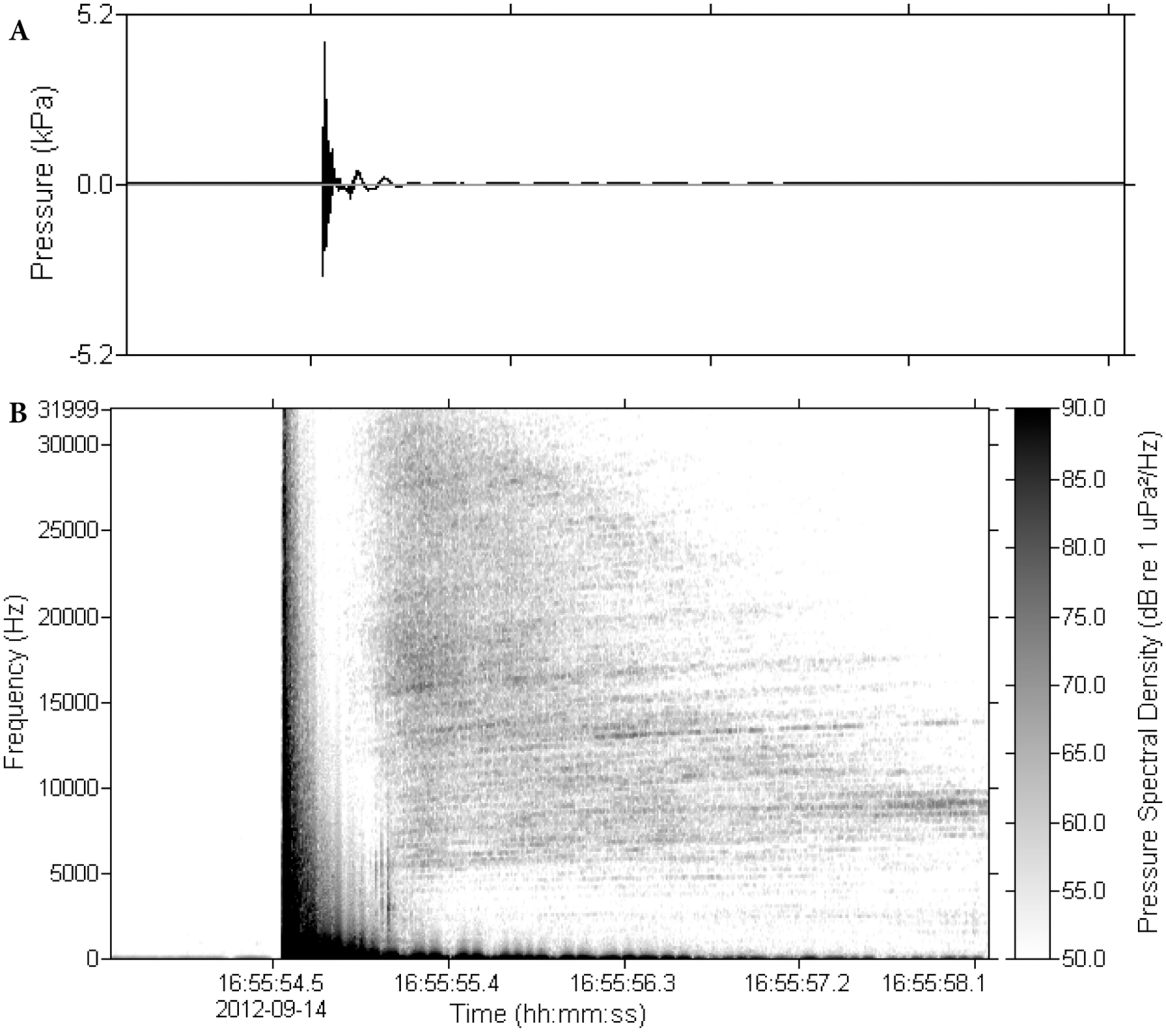
A representative sound recorded at cage 3. Top shows the time domain of the signal and the bottom shows the spectrogram. See Table 3 for details about the sounds at cage 3.

Two real-time systems, each consisting of an acoustic data acquisition and monitoring system (ADAMS; JASCO Applied Sciences Dartmouth, NS Canada) with a hydrophone and a laptop computer), were used to display and record acoustic data. The real time systems were used to sample the acoustic field during preliminary acoustic mapping and to monitor sound levels at the center of the two cages closest to the sound source when exposing fish to airgun sounds; thus providing quality control feedback for ensuring consistency of pulse levels and fish exposure. Four autonomous multichannel acoustic recorders (AMAR Mini; JASCO Applied Sciences) were used to acquire sound measurements for preliminary acoustic mapping and to monitor sound levels at the three cages farthest from the sound source and at a control location. The autonomous recorders were attached to moorings and deployed at the locations of the cages. Average sound levels at each location are presented in Table 3 and the time domain and spectrum of a typical pulse at cage 3 is shown in Fig 4. As expected, there was slight variation in sound level from pulse to pulse, which was < ±1 dB at all locations except Cage 2. The averages in Table 3 are for all shots and were recorded simultaneously at all locations. That Cage 2 had greater variation than the other sites suggests that it was at a near-field location where the vector sums from the individual airgun pulses were sensitive to small changes in phase; including time-delays from surface and bottom reflections.

**Table 3:** Average Sound Pressure Levels Measured at the Different Cages.

Cage Number	Distance from Airgun Array to Center of Cage (m)	Peak SPL (maximum) (dB re 1 μPa)	Peak-SPL (Peak-) (dB re 1 μPa)	SEL_ss_ (dB re 1 μPa^2^·s)	rms SPL (dB re 1 μPa)
1	0*	231 ± 0.8	225 ± 0.9	205 ± 0.4	225 ± 0.7
2	6.25	223 ± 1.4	221 ± 3.8	199 ± 2.9	215 ± 2.7
3	14.75	216 ± 0.5	212 ± 0.7	193 ± 0.7	206 ± 0.9
4	21	215 ± 0.5	211 ± 0.4	192 ± 0.5	205 ± 0.4
5	33.75	206 ± 0.9	206 ± 0.4	187 ± 0.4	199 ± 0.4
Control**	160 south	139 ± 7.7	138 ± 7.7	125 ± 4.0	105 ± 4.3

The ambient noise level in the control cage was recorded when fish were present, but without airgun pulses.

Number of samples at cage locations 1 to 5 = 64; number of samples at Control = 13.

* Cage 1 was just above or below the airgun array, depending upon species.

** Sound levels at the control cage represent ambient noise levels in the lake.

Peak SPL = peak sound pressure level whether positive or negative; Peak-SPL = peak negative sound pressure level.

SELss = Single strike sound exposure level.

### Necropsy

Once returned to the hatchery, fish were monitored every 12 h for 7 days post-exposure for distress or significant tissue damage. Procedures were to euthanize any animals that were showing distress-related behaviors such as swimming on their sides, staying at the surface for more than 5 minutes (both species do come to the surface for periods of time as part of their normal behavior), showing abnormal swimming patterns, or other abnormal behaviors. At no time did any fish show any kind of distress, and all animals survived the post-exposure period. They were then euthanized, refrigerated for an average of about 15 h and necropsied. Necropsy was done by a group of investigators who were trained prior to the study to ensure uniform methods and results following procedures developed and validated for studies of impacts of simulated pile driving and water guns on fish [13, 15, 16, 18, 19, 23, 24] and so will only be briefly summarized here.

### Necropsy Procedures

Fish were euthanized by being deeply anesthetized to a point of termination of respiration using buffered 750 ppm tricaine methanesulfonate (Western Chemical, Ferrndale WA, USA) in aerated hatchery water at 13.9°C. Fish were considered to have been euthanized once they showed no opercular movements for 10 min.

Following euthanasia, each fish was dip netted from the anesthetizing solution, had excess water removed by patting with a paper towel, wrapped in paper, labeled with time and day of refrigeration, and placed on a shelf in a walk-in cooler at 3.3°C. Refrigeration allowed for much more controlled necropsy procedures by allowing investigators to euthanize fish within a few minutes of one another in the evening and then dissect the following day. This strategy substantially reduced the time required for fish processing by investigators in any one day. After about 15 h of refrigeration the fish were removed from the cooler for necropsy.

Necropsies were conducted without those doing them knowing the exposure conditions of the fish they were dissecting. After recording tag data and measuring and weighing, the fish was placed into a dissecting tray and opened using surgical scissors starting at the vent (cloaca) and cutting anteriorly, ending at the pericardium (S2 Figs). Great care was taken to ensure that the excision was medial and superficial and that organs of the peritoneum were not injured during the cutting process.

Fish were immediately evaluated to assess bruising, hemorrhaging, and swim bladder condition. After the internal organs and body wall were evaluated, these organs were carefully removed or shifted to the side to complete a more thorough examination of the swim bladder and kidneys. Photographs were taken of all tissues dissected and the internal condition of swim bladder, liver, and kidney as well as any additional trauma that was noted.

### Statistical Analysis

The experimental units in the study were individual cages with multiple fish inside. Each cage represented a binomial sample of *n*_*i*_ fish, of which *x*_*i*_died or had mortal injury. There were five sound level classes (represented by Cages 1 to 5), with the sound level decreasing with distance from the sound source (Table 3). Each cage of fish received the sound generated by a single pulse of the seismic array so that each cage of fish had a separate measure of sound exposure. Two sound covariates were used as independent variables to assess the relationship between sound level (exposure) and death/mortal injury (response): peak negative sound pressure level (Peak- SPL) and sound exposure level (SEL). There also were controls where fish received the same handling as treatment fish except for exposure to sound. Because observations of death/mortal injury among control fish were made, an Abbott’s adjustment [25] to the treatment fish was necessary.

The Abbott’s adjustment [25] is based on the assumption that surviving handling (i.e., control survival) is independent of surviving the treatment, such that:

$$E\left(S_{i}\right)=S_{c}S_{t}{}_{{}_{i}}$$

where

*S*_*i*_ = observed survival of test fish exposed to handling and treatment *i*

*S*_*c*_ = probability of surviving handling (i.e., control survival)

*S*_*Ti*_ = probability of survival for fish exposed to a treatment *i*

Data analysis was therefore based on numbers of test fish that were alive and healthy (i.e., *n*_*i*_*—x*_*i*_) using generalized linear models [26] with a binomial error structure and log-link. The control fish provided an independent estimate of *S*_*c*_ in the analysis. Analysis of deviance (ANODEV) was used to test hypotheses based on acquired data.

## Results

**Fig 5:**
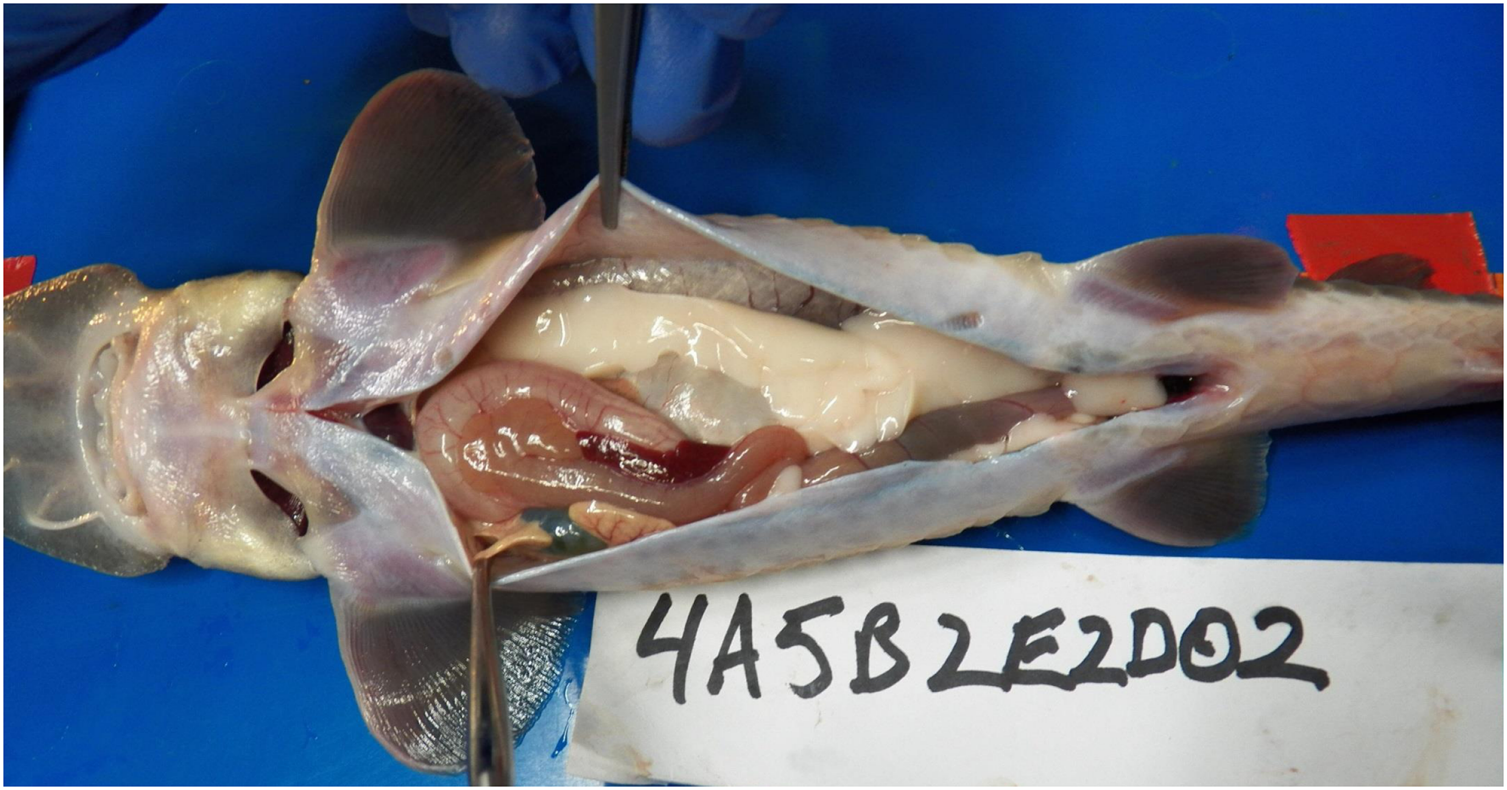
Examples of necropsied tissue. Figure shows a ventral dissection of a pallid sturgeon 4A5B2E2D02after exposure at a of 0.61m and a depth of 6.09 m. All tissues are healthy and no different from controls.

Test fish were exposed to sound in cages located horizontal distances of 0, 6.25, 14.75, 21, or 33.75 m from the anchored airgun array. The peak negative sound pressure level (peak-SPL) at Cage 1 (closest to the array) was approximately 230 dB re 1 μPa and the sound in Cages 2 through 5 had negative peak sound levels of approximately 221, 212, 210, and 205 dB re 1 μPa, respectively (Table 3). The experimental units were individual cages containing fish since each cage of fish was deployed separately and exposed independently to a single acoustic pulse from the seismic array (Table 3). A summary of the types of injuries encountered and the number of fish showing each injury is in Table 4. Fig 5 shows the dissection of a pallid sturgeon, where, as typical of all animals studied, there was no damage to any internal tissues. Similar results were found for controls.

**Table 4:** Summarized Injuries for Both Species.

Species	Distance (m) from Source	Total Individuals	Mean Fork Length (mm)	Mean Mass (g)	Hematoma on Muscle Wall	Ruptured Swim Bladder	Bruised Swim Bladder	Deflated Swim Bladder (no Ruptures)	Kidney Trauma	Rena Edema	Fatal Injuries
Sturgeon	Control	15	411.0	211.5	0	0	2	1	3	14	3
	0	15	423.3	239.5	0	0	4	1	1	12	1
	6.25	15	411.6	215.4	0	0	3	1	4	14	3
	14.75	15	415.7	224.1	0	0	1	1	3	14	2
	21	15	413.7	231.1	0	0	1	1	3	15	3
	33.75	15	416.4	232.2	0	0	2	2	2	13	2
Paddlefish	Control	10	462.8	346.7	1	0	1	1	3	4	1
	0	11	463.3	346.7	1	0	1	0	4	8	4
	6.25	10	471.1	358.3	0	0	2	0	1	4	0
	14.75	8	472.7	363.3	1	0	2	0	1	3	1
	21	10	474.0	366.7	2	0	2	1	2	6	2
	33.75	10	472.2	363.3	1	0	1	0	1	10	1

### Pallid Sturgeon

No pallid sturgeon mortalities coincident with sound exposure occurred and no fish died during the 7-day holding period. Damages that could have resulted in ultimate death (see [18–20, 24]) were recorded from fish exposed to sound and in controls (Table 4). Consequently, the analysis of the experimental data required adjustment for control effects.

An R x C contingency table (Table 5) displays the raw counts for the five different treatment groups (cage distance 1 through 5) plus controls, after pooling across replicates. The observed proportions of fish with mortal injuries among the treatments ranged from 0.0833 to 0.2143. The control fish had an observed mortal injury proportion of 0.1538. Pooling across the five treatment groups, the observed proportion of mortal injuries was 0.1549, which was nearly identical to the control rate. The R x C contingency table found no difference in proportions of mortal injury among the six groups of fish $\left(P\left(\chi_{5}^{2}\geq 1.1893\right)=0.9461\right)$. Analysis of deviance (ANODEV) found no difference in the rate of mortal injury between the control and treatments pooled (*P*(*F*_1,27_ ≥ 0.0001) = 0.9924) or individually: (*P*(*F* ≥ 0.2017) = 0.9572). In addition, none of the five test groups had significantly higher rates of mortal injury than the controls (*P* ≥ 0.3554).

**Table 5:** Counts of Observed Mortal Injury by Treatment Group (proportion in parentheses) for Pallid Sturgeon.

Observation	Treatment Distance (m) and Control					
	0	6.25	14.75	21	33.75	Control
Alive and healthy	11	11	13	12	13	11
	(0.9167)	(0.7857)	(0.8667)	(0.8000)	(0.8667)	(0.8462)
Mortal injury	1	3	2	3	2	2
	(0.0833)	(0.2143)	(0.1333)	(0.2000)	(0.1333)	(0.1538)

Treatment Groups (cages) 1 through 5 are in order of increasing distance from sound source. Chi-square test of homogeneity was not significant $\left(P\left(\chi_{5}^{2}\geq 1.1873\right)=0.9461\right)$.

**Fig 6:**
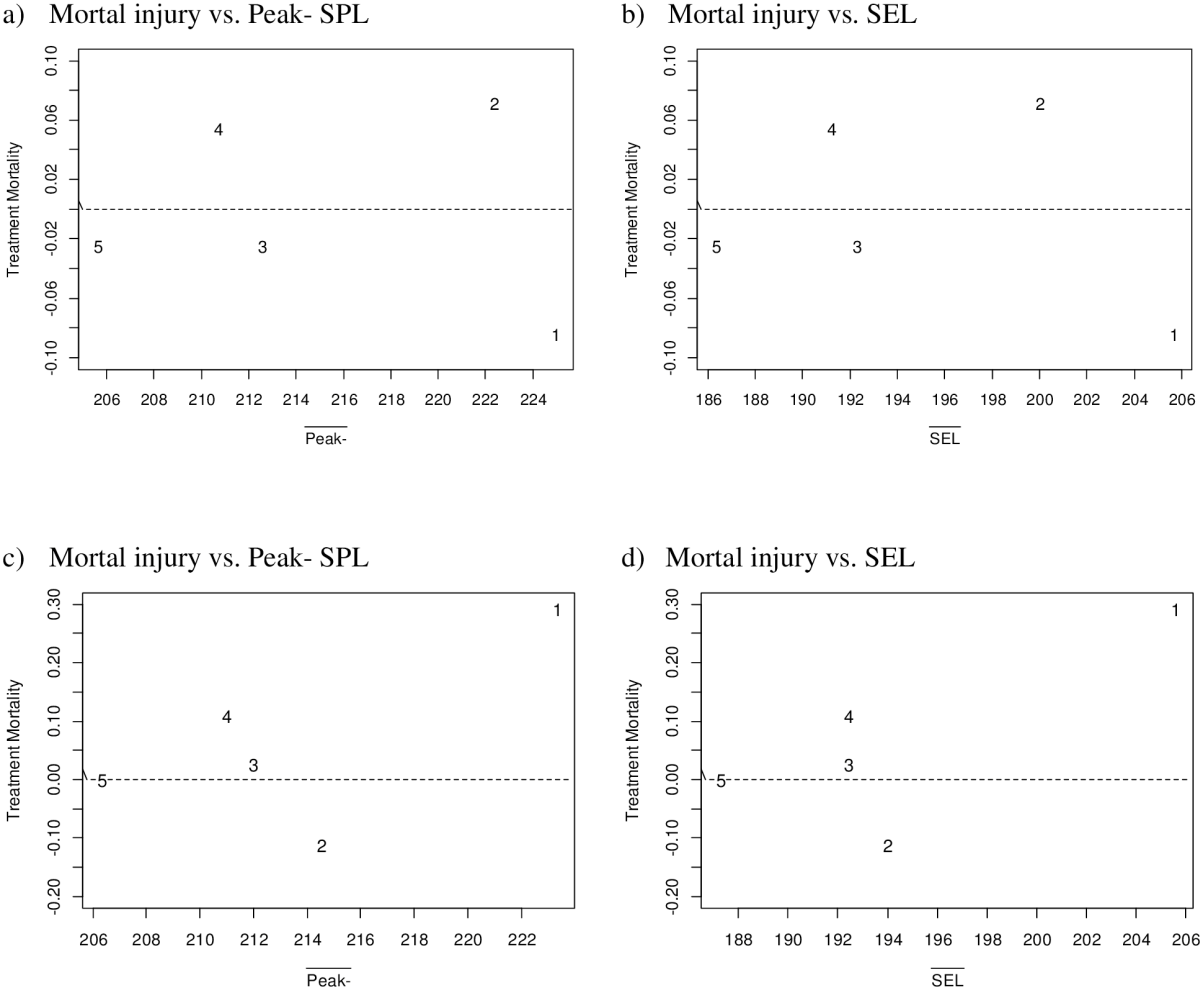
Scatterplots of observed rates of mortal injury after correction for control rates. Data plotted against: a) peak negative sound pressure level (Peak- SPL) and b) sound exposure level (SEL) for pallid sturgeon and c) peak negative sound pressure level (Peak- SPL) and d) sound exposure level (SEL_ss_) for paddlefish. Data were pooled over replicates and exposure levels averaged. Treatments 1 to 5 are in order of increasing distance from sound source. (Peak- SPL in units of dB re 1 μPa; SEL_ss_ in units of dB re 1 μPa^2^·s.).

ANODEV also was used to test whether there was a significant relationship between the level of sound exposure and the rate of mortal injury. No relationship was found between the peak negative sound pressure level (Peak- SPL) and the rate of mortal injury (*P* = 0.9987), nor between sound exposure level (SEL) and the rate of mortal injury (*P* = 0.9914). Plots of the observed rates of mortal injury after correcting for controls illustrate no pattern for either Peak- or SEL (Fig 6a and 6b).

Results of the analyses suggest that for the SELs tested there was no additional effect on mortality or mortal injury to pallid sturgeon from exposure to the impulsive sound generated by the airgun array.

### Paddlefish

Paddlefish experimental details were the same as for pallid sturgeon except the study consisted of three blocks each, in turn, consisting of five treatment samples and one control sample. The observed proportions of fish with mortal injuries (no mortalities observed) among the treatments ranged from 0.0 to 0.3636 in a non-monotonic pattern. The overall proportion of mortal injuries among treatment fish was 0.16. The control fish had an observed proportion of 0.10 with mortal injury. The R x C contingency table found no differences in proportions with mortal injury among the six groups of fish $\left(P\left(\chi_{5}^{2}\geq 6.5062\right)=0.2600\right)$.

ANODEV found no difference in the rate of mortal injury between the controls and all treatments pooled (*P*(*F*_1,16_ ≥ 1.775) = 0.6791 or individually (*P*(*F*_5,16_ ≥ 1.0829) = 0.4176) (Table 6). In addition, none of the five test groups had significantly higher rates of mortal injury than the controls (*P* ≥ 0.1167). The only treatment group that approached showing significantly higher mortal injury than the control group was the group in Cage 1, treatment 1 (*P* = 0.1167) with an observed mortal injury proportion of 0.3636.

**Table 6:** Counts of Observed Mortal Injury by Treatment Group (proportion in parentheses) for Paddlefish.

Observation	Treatment Distance (m) and Control					
	0	6.25	14.75	21	33.75	Control
Alive and healthy	7	11	7	8	9	9
	(0.6364)	(1.0000)	(0.8750)	(0.8000)	(0.9000)	(0.9000)
Mortal injury	4	0	1	2	1	1
	(0.3636)	(0.0000)	(0.1250)	(0.2000)	(0.1000)	(0.1000)

Treatment groups (cages) 1 through 5 are in order of increasing distance from sound source. Chi-square test of homogeneity was not significant $\left(P\left(\chi_{5}^{2}\geq 6.5062\right)=0.2600\right)$.

The ANODEV was used also to test whether there was a relationship between the level of sound exposure and the rate of mortal injury. Neither peak negative sound pressure level (Peak- SPL) (*P* = 0.6230) nor sound exposure level (SEL_ss_) (*P* = 0.6077) was related to the rate of mortal injury. Tests of positive relationships would have *P*-values of 0.3115 and 0.3039, respectively. Plots of the observed rates of mortal injury corrected for controls illustrate no definitive pattern in mortal injury for either Peak- or SEL (Fig 6c and 6d).

The analyses provided no definite evidence of increased mortality or mortal injury to paddlefish subjected to sound exposure. There is marginal evidence (*P* = 0.1167) that there were elevated rates of mortal injury at the closest treatment level 1 with the fish exposed to an average Peak- SPL of 223.3 dB re 1 μPa. However the small sample sizes make determining significant effects difficult at the individual treatment level.

## Discussion

The single pulse exposure paradigm used in this study was selected because it provided the closest simulation of the exposure to impulsive sound wild fish are expected to experience during seismic exploration of Lake Sakakawea or similar lacustrine environments. In such a seismic study, the vessel carrying the airgun moves along transects where a single pulse is generated by the airgun array at preplanned pulse points. After a pulse is completed, the vessel moves some distance (often on the order of 100 m) to the next pulse point. The distance traveled by the airgun vessel would likely ensure that if a fish were exposed to two pulses, one pulse would usually be higher in energy than the other. Therefore any observed effect could be assumed to primarily be a consequence of the higher energy exposure. Thus, in the present experiment, it was decided that a single pulse would be appropriate to simulate the effective sound level to which fish would likely be exposed during an actual seismic survey.

The initial goal in the experimental design was to develop a dose-response function whereby the levels of sound received by fishes at different distances from the source could be quantitatively related to the response of the fishes to the sound exposure, in terms of mortality and mortal injury at exposure or during or within 7 days of exposure. However, because no statistically significant response of test fish to different levels of seismic sound was found, the results did not provide data that could be used to derive a dose-response function. Even at the highest sound levels, there was no mortality or mortal injury that was significantly different between controls and the fish exposed to the highest sound energy.

The results do not support the hypothesis that there would be mortality of fish exposed to the impulsive airgun sound, at least at peak received sound pressure levels as high as 231 dB re 1 μPa (208 dB re 1 μPa^2^·s SEL_ss_, Table 3). The evaluation of mortality and mortal injury occurred over 7 days post-exposure. At the time the study was completed on day 7 post exposure the rate of mortal injury did not differ statistically between exposed and control fish. Thus, it may be concluded that the sound levels from the seismic airgun used in this study (as used for actual seismic surveys in lakes or rivers) were not sufficiently high, in terms of negative overpressure magnitude, to cause statistically significant lethal injury either immediately or within 7 days of exposure in sturgeon and paddlefish. It is important to emphasize that this study focused on assessment of mortality and mortal injury caused by exposure of juvenile fish to impulsive sound generated by a small array of seismic airguns. The fish studied were caged to ensure that they were exposed to known sound levels.

### Extrapolation of Data to Fishes of Different Sizes

The length of all animals of each species used in the study was within one standard deviation of one another in order to eliminate size as a variable in the results. There may be some concern that the results cannot be extrapolated to larger or smaller fish. There are no available studies that have examined effects of impulsive sounds, including those produced by seismic airguns, on fishes of different sizes. The one potentially relevant study that did examine effects of sounds from underwater explosions on fishes of different sizes showed that as animals get larger it takes higher sound levels, on the order of 5 dB increase in SEL for each kilogram increase in fish mass, to show damage [27]; also see analysis in [21, 28]. However, the earlier study used explosive energy and not sound. This difference may well explain why opposite results were found in a study on effects of simulated pile driving sounds on at least one fish species [13]. Studies that observe the effect of exposure to intense sounds on fish over a range of sizes are needed to provide information necessary to characterize the expected differential in physiological response of fish of different sizes and age classes to impulsive sound.

In the case of both pallid sturgeon and paddlefish, animals were spawned and raised in the hatchery. It is possible that hatchery-raised animals could have a different hardiness or be physiologically “different” than wild animals, and thus wild pallid sturgeon and paddlefish might show physiological effects even though these were not seen in the hatchery animals. It is also possible that body fat noted during necropsy could have been protective in hatchery animals by insulating tissues surrounding the swim bladder from its movements in the impulsive sound field. Such “insulation” might prevent the swim bladder walls from striking and damaging nearby tissues [17, 21, 23, 29, 30]. Alternatively other conditions such as increased adiposity and renal edema noted during the necropsies could have suggested these fish would be less healthy than those free ranging individuals. Necropsy was challenging, as to view all essential organs the body fat would need to be moved aside or physically removed to best view kidneys for example. Additionally since no positive control for barotrauma was available, each fish was carefully handled, and deliberately necropsied by staff that were blind to treatment and questioned any abnormality.

### Acclimation to Depth

Fishes use their swim bladders to manage their buoyancy at different depths by adding or removing air as they change depth. Some species do this by gulping air at the surface of the water before they descend (physostomous species) or use a special gland as part of the swim bladder to pump air from the blood into the chamber (physoclistous species) [29]. In either case, if the swim bladder is not properly inflated at the depth of the animal the fish cannot maintain its position in the water column, making it expend additional energy.

More importantly for this study, if the swim bladder is not properly inflated, the walls are not in contact with surrounding tissues. Moreover, when the animal is exposed to an impulsive source the walls do not move with the same amplitude or speed as they do in a fish with a normally inflated swim bladder. Thus, a fish that does not have proper swim bladder inflation for the depth at which it is exposed is less likely to show injuries than would a fish in which the swim bladder is properly inflated. While a number of fish were found with deflated gas bladders, no fish were recorded having a ruptured gas bladder (Table 4).

It is not clear whether the fish used in the study were physiologically acclimated to the exposure depth or not. The fish were handled three times, including being paced in exposure cages, before being lowered to depth as soon as they were placed in the cages and then exposed to sound within about a minute of reaching depth. As a consequence, the physostomous pallid sturgeon and paddlefish may not have had sufficient time at the surface to gulp the air they would need to have a properly filled swim bladder at 2-m depth (approximately 120.9 kPa absolute pressure [Atmospheric pressure at sea level is about 101.3 kPa]) in the case of the paddlefish and 6 m (approximately 160.2 kPa absolute pressure) for the pallid sturgeon. While there is a possibility that some test fish may have been able to gulp some air prior to submergence, no data were acquired to test the assumption that test fish did or did not have inflated swim bladders prior to exposure to sound. However, it is certain that test and control fish were handled precisely the same and had the same transport experiences. The only difference between them was exposure to seismic sound. The number and range of injuries were statistically no different for the sound exposed and control fish (Tables 4 and 5), indicating that the state of acclimation of test and control fish was the same.

### Implication of Results for Other Seismic Studies

The results of this study show that pallid sturgeon and paddlefish with body mass on the order of 200 to 400 g exposed to a received single impulse as high as 231 dB re 1 μPa SPL _peak_ and 208 dB re 1 μPa^2^·s SEL_ss_ did not die immediately or within 7 days of exposure, and that the probability of mortal injury did not differ between exposed and control fish. Other seismic exposures (e.g., marine) will differ in many ways from the exposure used here, including in number of sounds to which fish are exposed, water depth, source size, etc. Moreover, since pallid sturgeon and paddlefish have body shapes that differ considerably from other fishes, and since this work was done in relatively shallow water, extrapolation to other species and other environments requires some caution.
